# The impact of social determinants of health on diabetic gastroparesis: a retrospective analysis

**DOI:** 10.3389/fpubh.2025.1695472

**Published:** 2025-10-07

**Authors:** Ioana Soare, Nicoleta Andreea Tudose, Claudia Simona Stefan, Roxana Elena Mirică

**Affiliations:** ^1^Department of Social Insurance Medicine, Titu Maiorescu University, Bucharest, Romania; ^2^Oncology Department, Elias Hospital, Bucharest, Romania; ^3^University Dunarea de Jos, Galati, Romania; ^4^Faculty of Medicine, Carol Davila University of Medicine and Pharmacy, Bucharest, Romania; ^5^Regina Maria, Private Healthcare Network, Bucharest, Romania

**Keywords:** type 2 diabetes mellitus, diabetic gastroparesis, social determinants of health, obesity, low income

## Abstract

**Objectives:**

This study explores the importance of social factors, Social Determinants of Health (SDOH), particularly poverty, on patients with type 2 diabetes mellitus (T2DM), who developed gastroparesis. The analysis aimed to correlate social variables such as income, education, occupation, and loneliness with clinical outcomes and their association with clinical outcomes and healthcare utilization, using hospitalization frequency and symptom burden as proxy indicators of quality of life.

**Methods:**

This retrospective observational study analyzed a subgroup of 50 patients with diabetic gastroparesis, selected from a larger cohort of 250 patients diagnosed with gastroparesis. Diabetic gastroparesis was confirmed via gastric scintigraphy. Demographic, clinical, and social variables were analyzed, and data collection was facilitated through a structured instrument using the software EpiInfo.

**Results:**

In this retrospective observational study, the majority of patients were obese females over 60 years of age, retired, and residing in rural areas. Loneliness emerged as a significant aggravating factor. Low income was associated with increased complications, frequent hospitalizations, and higher overall healthcare expenditure.

**Conclusion:**

Diabetic gastroparesis is an underdiagnosed complication of T2DM, frequently worsened by social vulnerability. In this study, low income, rural residence, obesity and loneliness were identified as key social determinants modulating disease severity and healthcare utilization. Greater emphasis on addressing these SDOH is necessary to optimize outcomes and reduce hospitalization rates, insurance expenses, and overall healthcare burden (used herein as proxy indicators of reduced quality of life).

## 1 Introduction

Recent advances in medicine, including integrating artificial intelligence and large-scale epidemiologic data into clinical practice, has underscored the crucial role of Social Determinants of Health (SDOH) in shaping health outcomes. The World Health Organization has emphasized the importance of SDOH since 2008, providing an operational framework in 2024. Factors such as socioeconomic status, education, occupation, and social isolation have been increasingly recognized as central to disease management and prognosis (1).

Professor Rachel Rowe of the School of Population Health, Australia, published an article on the Social Determinants of Health in the journal *Big Data & Society,* in which she stated: “In recent years, the claim that around 80 percent of contemporary health issues are attributable to social factors has become a mantra at digital health technology conferences” (2).

**Figure 1:**
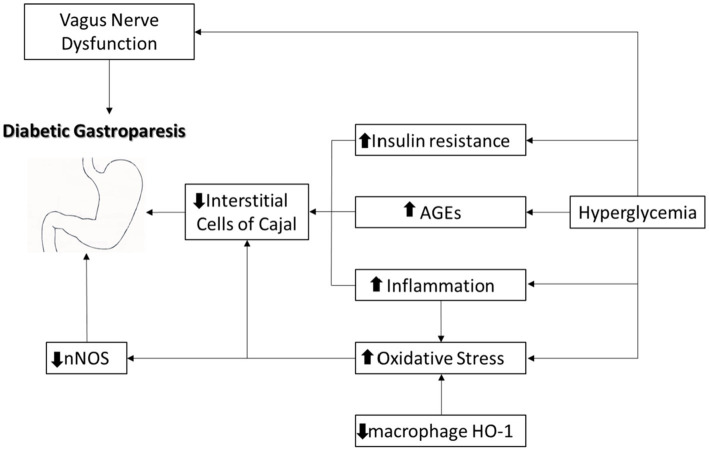
Main pathophysiological mechanisms associated with diabetic gastroparesis development. AGE—advanced glycation end-product (32).

Diabetes mellitus (DM) is the most common condition associated with gastroparesis (3). Diabetic gastroparesis is a chronic complication of long-standing and poorly controlled diabetes, often associated with autonomic neuropathy, smooth muscle cell dysfunction, and damage caused by the reduction in interstitial peacemaker Cajal cells (4–6), at the level of the myenteric plexus, and through the appearance of fibrosis (7–9). It is characterized by delayed gastric emptying in the absence of mechanical obstruction and results in significant morbidity (2, 4, 10). A recent alternative theory highlighted the role of oxidative stress in the pathogenesis of diabetic gastroparesis (11, 12) through increasing the number of macrophages and amplifying heme oxygenase-1 (HO1), while causing a selective decrease in protective CD 206-positive macrophages (13) (Figure 1).

Acute hyperglycemia (glycemia values above 200 mg/dL) contributes to delayed gastric emptying, which is often reversible upon glycemic normalization, influencing the gastric electrical activity (14) by relaxing and increasing the sensitivity of the proximal stomach and suppressing antral and pyloric contractions. On the other hand, in the presence of chronic hyperglycemia, gastroparesis does not improve, even with glycemic control (15–17). Hypoglycemia tends to accelerate gastric emptying in patients with gastroparesis (18).

Despite its clinical importance, it remains underdiagnosed and undertreated, particularly in socioeconomically disadvantaged populations.

This study investigates the intersection between diabetic gastroparesis and SDOH, with a focus on poverty, education, and social isolation, aiming to elucidate their impact on the disease burden, symptomatology, and healthcare utilization.

## 2 Materials and methods

### 2.1 Study design and participants

A retrospective observational study was conducted using medical records from the archives of the medical departments of a Bucharest hospital over a one-year period, between February 2019 and February 2020. From a total of 250 patients diagnosed with gastroparesis, we selected 50 patients with scintigraphy-confirmed diabetic gastroparesis. From a total of 250 patients with a discharge diagnosis of gastroparesis in the archive, 50 patients were selected for this study because they met all inclusion criteria: diagnosis of type 2 diabetes mellitus and gastroparesis confirmed by gastric emptying scintigraphy, and availability of a complete clinical and social determinants dataset. The remaining patients were excluded due to absence of scintigraphic confirmation, non-diabetic gastroparesis etiologies, or incomplete social/clinical records that precluded reliable analysis.

### 2.2 Data collection

All patients provided informed consent. Data were systematically recorded using a structured form developed using the software EpiInfo (distributed freely by the WHO). The inclusion criteria included a diagnosis of type 2 diabetes and confirmation of gastroparesis via gastric emptying scintigraphy. The exclusion criteria included evidence of mechanical gastric outlet obstruction or concurrent malignancies.

The variables analyzed included demographics (age, sex, residence), clinical results (obesity, glycemic control, comorbidities, alcohol and tobacco use, endoscopy results), and social determinants (income level, employment status, self-reported loneliness).

Quality of life (QoL) was not directly measured using a validated patient-reported outcome instrument in this retrospective dataset. Instead, we used proxy indicators associated with QoL — specifically, the annual frequency of hospitalizations (≥2 hospitalizations/year was considered a marker of high healthcare utilization and impaired QoL) and the burden of upper-gastrointestinal symptoms (epigastric pain, nausea, vomiting, early satiety) recorded at admission. We acknowledge this as a limitation and discuss it accordingly.

### 2.3 Ethical considerations

The study was approved by the local ethics committee, and all procedures followed the Declaration of Helsinki principles.

### 2.4 Statistical analysis

Descriptive statistics were used to summarize the patient characteristics.

Data were entered and managed in Epi Info v7. Statistical analyses were performed using IBM SPSS Statistics v26 (IBM Corp., Armonk, NY) and validated in Epi Info.

- Continuous variables are presented as mean ± standard deviation (SD) or as median with interquartile range (IQR), and categorical variables as absolute numbers and percentages.
- Normality of continuous variables was assessed with the Shapiro–Wilk test.
- Group comparisons were performed using Student’s t-test for normally distributed continuous variables or Mann–Whitney U test for non-normally distributed variables.
- Categorical variables were compared using Pearson’s chi-square test; Fisher’s exact test was applied when expected cell counts were <5.
- Associations between variables were evaluated with Pearson correlation coefficients (for normally distributed variables) or Spearman rank correlation (for non-normally distributed variables).
- Logistic regression (univariate and multivariate) was used to analyze predictors of frequent hospitalizations (≥2 admissions per year). Variables with *p* < 0.10 in bivariate analysis were entered into the multivariate model. Model fit was evaluated using the Hosmer–Lemeshow test, and results were expressed as odds ratios (OR) with 95% confidence intervals (CI).
- All tests were two-tailed, and a *p*-value < 0.05 was considered statistically significant.

## 3 Results

The manuscript focuses on associations between SDOH and clinical outcomes and healthcare utilization in a subgroup of T2DM patients with scintigraphy-confirmed gastroparesis; claims about direct measurement of quality of life have been reframed to reflect proxy indicators used in this retrospective dataset. Of the 250 patients identified in the archive with a diagnosis of gastroparesis, 50 met the inclusion criteria (T2DM with scintigraphy confirmation and complete data) and were included in the analysis.

### 3.1 Demographic profile

**Figure 2:**
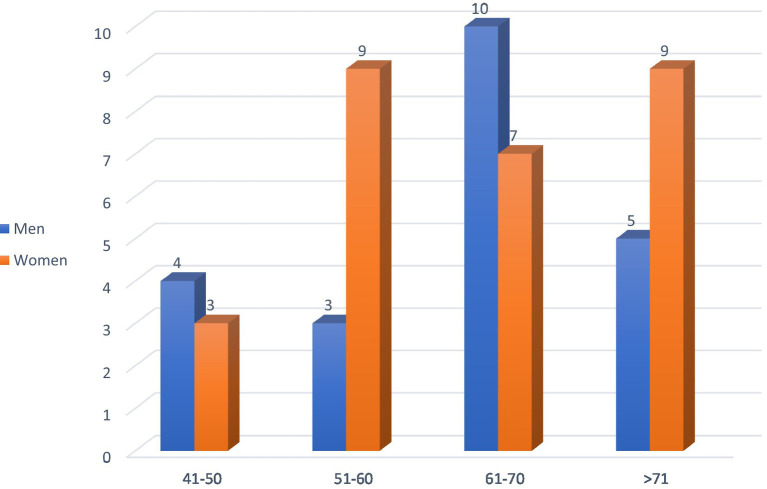
Gender distribution by age group.

**Figure 3:**
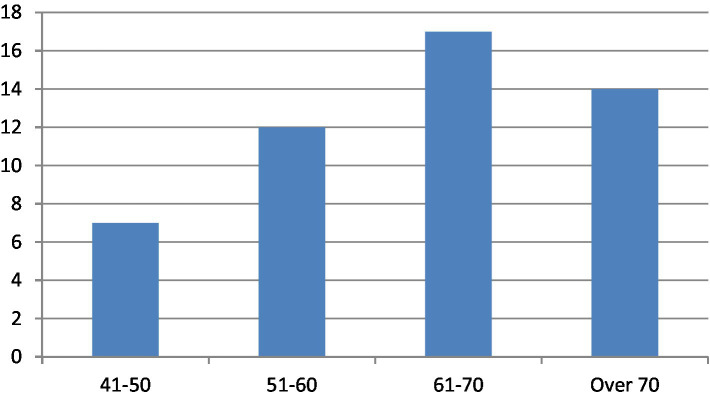
Age distribution of patients with diabetic gastroparesis. Most patients were aged 61–70 years.

Among the 50 patients with diabetic gastroparesis, the majority were women (56%) (Figure 2), and the patient ages ranged between 61 and 70 years (Figure 3). A significant majority (68%) were retired, with 32% still employed.

### 3.2 Social and lifestyle factors

In total, 50% of patients were classified as obese (BMI ≥ 30 kg/m^2^), and obesity was more prevalent among women. Chronic alcohol consumption was twice as common among males (32%) than females (12%), while cigarette use was similar across genders (males 28%, females 25%). Among the 50 patients with diabetic gastroparesis, the following comorbidities were present: hypertension (40%), dyslipidemia (36%), chronic kidney disease (14%), and cardiovascular disease (12%). Loneliness was frequently reported, particularly in older rural women. A notable proportion of patients reported social isolation, which correlated with suboptimal self-care, poor glycemic control, and frequent hospital admissions (Table 1).

**Table 1:** Key demographics and clinical features.

Variable	*n* (%)	*p*-value
Female	34 (68%)	—
Obesity (BMI ≥ 30)	25 (50%)	0.04 (F vs. M)
Alcohol consumption	16 (32%)	0.01 (M > F)
Smoking	14 (28%)	0.72
Loneliness (self-reported)	22 (44%)	—
Diffuse erosive gastritis	30 (60%)	0.002 (long DM duration)
≥2 hospitalizations/year	24 (48%)	0.03 (linked to loneliness)
Hypertension	20 (40%)	–
Dyslipidemia	18 (36%)	–
Chronic kidney disease	7 (14%)	–
Cardiovascular disease	6 (12%)	–

### 3.3 Endoscopic findings

Ninety-two percent of patients underwent upper digestive endoscopy. The most common findings were as follows:

- Diffuse non-specific gastric mucosal changes;
- Erosive and erythematous lesions;
- Hypertrophic mucosal changes.

### 3.4 Clinical presentation and impact of income on the symptoms

The most frequently reported symptoms were epigastric pain, nausea, vomiting, abdominal bloating, and altered intestinal transit. Less common symptoms included bowel irregularities, early satiety, and weight loss. Notably, the symptoms were often mild and non-specific, contributing to delayed diagnosis and management challenges.

Patients with low income (n = 36) had higher rates of all symptoms—epigastric pain, nausea, vomiting—but none reached statistical significance (Table 2).

**Table 2:** Symptoms by income group.

Symptom	Low income (*n* = 36)	Higher Income (*n* = 14)	*p*-value	OR (95% CI)
Epigastric Pain (%)	80%	64%	0.18	2.29 (0.67–7.77)
Nausea (%)	75%	57%	0.21	2.27 (0.66–7.79)
Vomiting (%)	65%	43%	0.15	2.44 (0.69–8.65)
Hospitalizations (mean/year)	2.7 ± 1.0	1.3 ± 0.7	0.005*	–

^*^Significant at *p* < 0.05.

### 3.5 Comparison by place of residence

Comparisons between rural (*n* = 30) and urban (*n* = 20) patients were performed using Student’s t-test for continuous variables (age, HbA1c, hospitalizations) and Pearson’s chi-square test or Fisher’s exact test for categorical variables (sex, obesity, income, education level). The socioeconomic analysis indicated that 78% of patients had low income, correlating significantly with increased hospitalization rates (*p* < 0.05). Rural residence was associated with higher rates of obesity (60% vs.35%, *p* = 0.03, chi-square test) and poorer glycemic control (mean HbA1c 8.5% vs. 7.3% in urban patients, *p* = 0.01, *t*-test). Rural patients had a higher mean age (66.2 ± 5.8 vs. 63.5 ± 6.1 years, *p* = 0.12, *t*-test); however, this was not statistically significant. Regarding educational attainment 17 (34%) had higher education (university degree), 25 (50%) had completed high school or vocational school, and 8 (16%) had only primary education. A higher proportion of patients with lower educational attainment were from rural areas (6 of 8 with only primary education) compared to urban areas (2 of 8). While educational level appeared lower among rural residents (6/30 vs. 11/20, *p* = 0.02, Fisher’s exact test), statistical analysis did not show a significant correlation with hospitalization rates or symptom severity, possibly due to the small sample size. Educational attainment, together with income level, occupational status, and social isolation, represents an important social determinant of health that may influence disease management and outcomes in patients with diabetic gastroparesis. Diabetes duration was stratified into two categories: <10 years and ≥10 years. Among rural patients, 40% had diabetes for less than 10 years and 60% for 10 years or more. In urban patients, 50% had diabetes <10 years and 50% ≥ 10 years (Table 3). Disease duration (<10 years vs. ≥10 years) did not differ significantly between rural and urban patients (*p* = 0.45, chi-square test). Longer disease duration was associated with higher hospitalization rates among rural patients (2.5 ± 1.1 vs. 1.4 ± 0.8, *p* = 0.02, t-test) and increased complications, consistent with the known association between long-standing diabetes, autonomic neuropathy, and gastroparesis (Table 3). These analyses demonstrate that rural residence is associated with poorer glycemic control, higher obesity prevalence, and increased healthcare utilization, highlighting the impact of social determinants on disease severity.

**Table 3:** Comparation between rural and urban patients.

Variable	Group 1: Rural (*n* = 30)	Group 2: Urban (*n* = 20)	*p*-value	95% CI of Difference (Rural - Urban)
Age (mean ± SD, years)	66.2 ± 5.8	63.5 ± 6.1	0.12	−1.1 to 6.3
Female (%)	70%	65%	0.68	−20 to 30%
Obesity (%)	60%	35%	0.03*	5 to 45%
HbA1c (mean ± SD, %)	8.5 ± 1.2	7.3 ± 1.0	0.01*	0.5 to 2.2
Low Income (%)	78%	60%	0.09	−3 to 40%
Education level (Primary/High School/Higher)	6/18/6	2/7/11	0.02*	–
Higher education	8 (27%)	9 (45%)	0.18	−10 to 45%
High school/vocational	15 (50%)	9 (45%)	0.74	−20 to 30%
Primary school	7 (23%)	2 (10%)	0.22	−5 to 30%
Disease Duration				
<10 years	12 (40%)	10 (50%)	0.45	−20 to 40%
≥10 years	18 (60%)	10 (50%)	0.45	−40 to 20%
Hospitalizations (mean/year)	2.5 ± 1.1	1.4 ± 0.8	0.02*	0.3 to 2.1

*Significant at *p* < 0.05.

Comparisons between rural and urban groups. Continuous variables are presented as mean ± SD and were compared using Student’s t-test when normally distributed or Mann–Whitney U test when not. Categorical variables were compared using Pearson’s chi-square test; Fisher’s exact test was used where expected cell counts were <5. *p*-values < 0.05 were considered statistically significant. The specific test used for each row is indicated in the rightmost column where applicable.

## 4 Discussion

Diabetic gastroparesis is a chronic condition characterized by delayed gastric emptying, which is often overshadowed by more prominent complications such as nephropathy, retinopathy, and cardiovascular disease. However, it significantly impairs the quality of life and complicates glycemic control, creating a vicious cycle.

Diagnosis begins by ruling out mechanical obstruction by performing an upper endoscopy, enteroCT, or magnetic resonance enterography (MRE) (4) and confirming delayed gastric emptying by scintigraphy, which remains the gold standard for diagnosis, but it is underutilized in routine practice (5). Delayed diagnosis is common due to the non-specific nature of the symptoms and a general underappreciation of gastric complications in diabetes care.

The management strategies include optimizing glycemic control (19), dietary adjustments (reducing the intake of products rich in fats and spices (20, 21) and increasing the amount of fiber), adequate hydration (22), and lifestyle changes (avoidance of alcohol, tobacco, and carbonated beverages) that decrease antral contractility and delay gastric emptying (23, 24).

The first step in the treatment of compensated diabetic gastroparesis (mild symptoms) is the optimization of glycemic control (since hyperglycemia delays/slows gastric emptying in patients with diabetes mellitus) (19). Incretinomimetics (Pramlintide) and GLP-1 (glucagon-like peptide-1) receptor agonists or analogues (Exenatide) should be avoided, as they exhibit the same effect. In contrast, oral antidiabetic agents such as dipeptidyl peptidase-4 inhibitors (Vildagliptin) do not affect gastric emptying (19). Other stages of gastroparesis management involve pharmacological therapy such as antiemetics and prokinetic agents and surgical methods in the case of severe forms (gastroparesis with gastric failure) with persistent symptoms (25). In addition, integrating diabetes-specific therapies such as GLP-1 receptor agonists or other pharmacologic regimens may influence gastric motility, and future studies should explore these effects in relation to social determinants of health.

Recent studies emphasize the critical role of Social Determinants of Health (SDOH) in shaping the outcomes of chronic noncommunicable diseases, including diabetes. Factors such as low income, limited education, social isolation, rural residence and comorbid conditions not only exacerbate disease progression but also increase hospitalization rates and reduce access to effective care.

The 5 domains of the framework of SDOH are the following: Health and Health Care, Economic Stability, Neighborhood and Built Environment, Education, and Social and Community Context. A 2021 review by the American Diabetes Association assessed single SDOH domains (26), toward the goal for health improvement among the population with diabetes (27). Levy et al. conducted a cross-sectional study in which the results highlighted high levels of SDOH barriers across all 5 domains, including significant levels of diabetes-related distress (26). The outcomes of another study conducted by Zhang et al. showed that SDOH have an independent effect on cardiovascular events in patients with diabetes (28).

In our study, the impact of social determinants of health (SDOH) on diabetic gastroparesis was analyzed beyond the most evident factors such as low income, rural residence, and social isolation. By including education, diabetes duration, occupation, and comorbidities, we provide a more comprehensive view of how social and clinical factors interact to influence disease severity and healthcare utilization. Education level appeared to correlate with patients’ ability to recognize symptoms, adhere to dietary and pharmacological recommendations, and navigate healthcare resources, highlighting this role as a potential modifier of disease management and self-care. With 17 patients having higher education, 25 with secondary or vocational schooling, and 8 with only primary education. The duration of diabetes, stratified as <10 years and ≥10 years, revealed that patients with longer disease histories had higher rates of gastroparesis complications, highlighting its role as a potential confounder. Occupational status, reflecting daily routines and access to care, further modulated disease management, while the presence of comorbidities—such as hypertension, dyslipidemia, chronic kidney disease, and cardiovascular disease—contributed significantly to hospitalization rates and impaired quality of life. These findings underscore that the progression and management of diabetic gastroparesis are determined by a complex interplay of socioeconomic, occupational, and clinical factors, and that addressing SDOH in a holistic manner is essential for reducing disease burden and optimizing patient outcomes.

The prevalence of gastric mucosal lesions supports the hypothesis that prolonged gastric stasis aggravates mucosal damage, often masked by mild symptoms.

Recent specialist articles revealed that gastroparesis affects approximately 18% of diabetes patients. Its incidence is estimated at approximately 5% in type 1 and 1% in type 2 diabetes (3). Although there is an association between gastroparesis and type 1 diabetes, it is frequently observed in type 2 diabetes due to incretin mimetics (glucagon-like peptides administered to patients with type 2 diabetes), which increase the risk of developing gastroparesis (28). A higher prevalence has been noted in urban environments (29), in those who have been diagnosed with diabetes for at least 10 years, and in older patients with type 2 diabetes who have comorbidities (4). The risk factors for diabetic gastroparesis include microangiopathic complications, obesity, female gender (30, 31), and associated psychiatric conditions. In Sweden, a study of 217,000 patients carried out over 7 years evaluated several social factors, including education, income, occupation, and loneliness, in terms of their ability to influence the quality of life of patients with type 2 diabetes; diabetic gastroparesis was more frequently associated with females, especially obese female patients.

In a cross-sectional study from 2024, Harrison et al. found that adverse SDOH including low income, lack of health insurance, and food insecurity were differentially associated with higher prediabetes prevalence among adolescents (32, 33).

In our study, patients over 60 years of age predominated in the group of patients with diabetic gastroparesis, 56% were female, and females with low incomes and rural residency were far more predisposed to developing complications than males from rural areas. In an urban environment, this discrepancy decreased. Equal proportions of females and males were smokers, although men were more likely to consume alcohol. In Romania, the number of males who smoke and chronically consume alcohol is higher than the number of females who engage in these practices. Although symptoms such as epigastralgia, nausea accompanied by vomiting, and intestinal transit disorders guide the diagnosis of gastroparesis, as diabetes mellitus deeply affects the innervation of the stomach, the intensity of these symptoms does not reflect the actual severity of the gastric damage. Thus, trivial and non-specific symptomatology can hide substantial gastric damage, which may be the reason why type 2 diabetes sometimes cannot be controlled by usual therapeutic means. The aggravation of gastroparesis in the studied patients was manifested by gastric lesions, which indicate uncontrolled diabetes with a long evolution. In specialized studies of patients with type 2 diabetes with upper digestive endoscopy, mucosal lesions with antral localization were the predominant type, which is explained by the fact that patients with diabetes have a higher risk of bile reflux. In 2016, a study conducted in Turkey on a group of 51 patients with type 2 diabetes, of whom 30 had gastroparesis proven by scintigraphy, found that under endoscopic examination, patients with gastroparesis had a higher frequency of erosive-type gastric lesions, a phenomenon explained by prolonged gastric stasis.

Our statistical analysis revealed that rural residence, low income, obesity, and poor glycemic control (HbA1c > 8.0%) were significant independent predictors of frequent hospitalizations. Patients from rural areas had significantly higher HbA1c levels and obesity rates, consistent with limited healthcare access and dietary differences. Although epigastric symptoms were more frequent among low-income patients, these differences did not reach statistical significance. However, their hospitalization rate was significantly higher, highlighting the burden of socioeconomic disparities in diabetes management. Logistic regression confirmed low income and poor glycemic control as strong predictors, aligning with the existing literature.

Thus, the first step in evaluating patients with type 2 diabetes, especially those with long-term evolution and multiple complications, must take into account the possibility of diabetic gastroparesis.

Importantly, addressing social determinants through multidisciplinary care, patient education, and community outreach can enhance outcomes.

### 4.1 Limitations

This study has several limitations. First, the sample size was relatively small and drawn from a single tertiary center, which may limit the generalizability of the results. Patients were recruited from a single tertiary care hospital, which may overrepresent more severe or complex cases, potentially introducing selection bias. Second, the cross-sectional nature of the study prevents causal inference between social factors and the severity of gastroparesis. Third, while we identified associations between social factors and disease characteristics, no statistical tests were applied due to the descriptive scope of the study. We acknowledge that QoL was not directly assessed by a validated questionnaire in this retrospective study; hospitalization frequency and symptom burden served as indirect proxy measures. Future prospective studies should include validated QoL instruments (e.g., ANMS GCSI-DD, SF-36, EQ-5D) to quantify the patient-reported impact of diabetic gastroparesis and to better link SDOH with patients’ lived experience. Future multicenter studies with larger samples and statistical modeling are warranted.

### 4.2 Perspectives and future research

The growing recognition of the importance of Social Determinants of Health (SDOH) in chronic disease management calls for a paradigm shift in both clinical care and public health strategy. In the context of diabetic gastroparesis, proactively addressing SDOH may prevent disease progression and reduce the healthcare burden.

From a preventive standpoint, future strategies should incorporate routine screening for SDOH in clinical settings using validated instruments, such as the PRAPARE (Protocol for Responding to and Assessing Patient Assets, Risks, and Experience screener) (34) or EPIC SDOH (35) tools. This could facilitate early identification of at-risk individuals and inform personalized care plans. Primary care teams, including social workers and patient navigators, should be integrated to support these patients holistically.

Community-level interventions—particularly in rural and low-income areas—may improve outcomes by addressing modifiable risk factors such as nutrition, glycemic control, and social isolation. Educational campaigns, social prescribing models, and mobile health units could be key tools in reaching vulnerable populations.

From a research perspective, the warranted future directions are as follows:

- *Longitudinal cohort studies* are needed to assess the cumulative impact of SDOH on the onset, severity, and healthcare utilization patterns of diabetic gastroparesis over time.
- *Randomized interventional trials* could evaluate the effectiveness of targeted SDOH interventions (e.g., nutritional support, transportation vouchers, social connection programs) in reducing hospital admissions and improving symptom control.
- *Predictive modeling using artificial intelligence* could aid in the early identification of high-risk patients by integrating clinical, biochemical, and social variables.
- *Cost-effectiveness analyses* are essential to justify policy changes and to demonstrate the financial benefits of incorporating social risk screening into diabetes care.
- *Development and validation of SDOH risk indices* tailored to the European diabetic population could enhance clinical decision-making and stratification of care.

Ultimately, bridging the gap between medical care and social support systems will be crucial to achieving equity and improving outcomes in diabetic gastroparesis and other chronic conditions.

## 5 Conclusion

Diabetic gastroparesis remains an underdiagnosed and underappreciated complication of type 2 diabetes mellitus, frequently misattributed to other causes, often overshadowed by more prominent macro- and microvascular complications that significantly impacts quality of life and healthcare costs. This study demonstrates that social determinants of health (SDOH)—including low income, social isolation, rural residence, and lower educational attainment—play a critical role in disease severity and healthcare utilization. Clinical factors such as obesity, comorbidities, and longer diabetes duration further exacerbate outcomes.

Addressing both medical and social dimensions through multidisciplinary care, patient education, and community-level interventions is essential to reduce hospitalizations and improve management in vulnerable populations. Future research should focus on prospective, multicenter studies and evaluate the impact of social and clinical factors on treatment response, guiding effective public health strategies for patients with diabetic gastroparesis.

## Data Availability

The raw data supporting the conclusions of this article will be made available by the authors, without undue reservation.
